# Mechanically driven strategies to improve electromechanical behaviour of printed stretchable electronic systems

**DOI:** 10.1038/s41598-020-68871-w

**Published:** 2020-07-21

**Authors:** Donato Di Vito, Milad Mosallaei, Behnam Khorramdel, Mikko Kanerva, Matti Mäntysalo

**Affiliations:** 1 Faculty of Engineering and Natural Sciences, Tampere University, Tampere, Finland; 2 Faculty of Information Technology and Communication Sciences, Tampere University, Korkeakoulunkatu 3, 33720 Tampere, Finland

**Keywords:** Electrical and electronic engineering, Theory and computation, Electronic devices, Polymers, Structural materials, Mechanical properties, Techniques and instrumentation

## Abstract

Stretchable electronics promise to extend the application range of conventional electronics by enabling them to keep their electrical functionalities under system deformation. Within this framework, development of printable silver-polymer composite inks is making possible to realize several of the expected applications for stretchable electronics, which range from seamless sensors for human body measurement (e.g. health patches) to conformable injection moulded structural electronics. However, small rigid electric components are often incorporated in these devices to ensure functionality. Under mechanical loading, these rigid elements cause strain concentrations and a general deterioration of the system’s electrical performance. This work focuses on different strategies to improve electromechanical performance by investigating the deformation behaviour of soft electronic systems comprising rigid devices through Finite Element analyses. Based on the deformation behaviour of a simple stretchable device under tensile loading, three general strategies were proposed: local component encapsulation, direct component shielding, and strain dispersion. The FE behaviour achieved using these strategies was then compared with the experimental results obtained for each design, highlighting the reasons for their different resistance build-up. Furthermore, crack formation in the conductive tracks was analysed under loading to highlight its link with the evolution of the system electrical performance.

## Introduction

Soft electronics are designed to overcome the general limitations of conventional electronic systems, that is the possibility to subject these systems to high levels of deformation while maintaining their electrical integrity^1,2^. Devices created with this purpose find application in a wide variety of fields, such as fitness monitoring and health care systems, as well as conformable injection-moulded structural electronics^3^. The aforementioned applications require the electrical system to deform together with the underlying materials: especially for health-patch applications, this means that such electrical devices have to seamlessly adapt to human skin deformations^4^, whose deformation levels can be highly heterogeneous^5^. These reasons make it difficult to restrict the working conditions for this set of systems^6^, while, on the other hand, they encourage the request for materials that have mechanical properties similar to the human skin, in order to improve comfort to the user and reduce obtrusiveness of such electrical systems^7,8^.

Stretchable electronic systems generally comprise several components, where each specifically addresses certain functions. These components typically are miniaturized functional modules such as batteries, sensors, radio transmitters and receivers, that are connected by stretchable interconnects. Structures that find vast application as interconnects for stretchable electronics are thin, metallic bi- and tri-dimensional structures, that yield promising results in terms of stability and maximum elongation at electrical failure^9,10^ by employing complex geometries for the interconnects such as zigzag, multi-track pattern, helical, and origami-inspired shapes^11–13^ and horseshoe designs^14–16^. These strategies employ mechanical phenomena such as local buckling and post-buckling behaviour to allow the conductive structure to deform out of the plane defined by the substrate underlying, thus increasing the range of deformability without reaching mechanical failure, thus allowing them to maintain electrical conductivity of bulk metallic conductors even at high overall deformation levels^15^. However, application of these processes significantly increases the complexity of the fabrication.

The need to find materials that show conductive properties comparable with metals, while having deformability and stiffness that are closer to polymers and elastomers, resulted in the employment of specific fabrication processes to create micro- and nanostructured elements from metallic materials and the development of new intrinsically conductive materials with better electrical and mechanical properties^17^.

Materials related to this strategy are mainly micro or nanocomposites constituted by different polymers featuring low elastic modulus together with conductive fillers, which increase the electrical conductivity of the medium. Most of these micro and nanocomposites are generally constituted by a conductive material, such as Ag μm- or nm-sized particles or by different carbon-based conductors, such as CNTs and graphene, together with a polymeric matrix^2,18–22^. These classes of conductive composites offer high flexibility of properties and many advantages over the thin metallic films that are generally used in this field. Moreover, they can be printed on top of the desired substrate, which can range from thermoplastic polyurethanes (TPUs)^8,23^ and polydimethylsiloxanes (PDMS)^24,25^ to textiles^26,27^. However, conduction in these materials is based on deformation mechanisms that are radically different from the ones available for thin metallic films: in fact, for these materials conduction is carried through the filler particles, that are in contact with each other and act as rigid inclusions, while the polymeric binder typically carries the deformation transferred from the substrate. Because of the heterogeneity of the material and of its peculiar conduction properties and processes, the definition of electrical failure as well as the electrical resistance build-up are properties difficult to extensively characterize and model. Due to the presence of the polymeric binder, the overall elastic modulus of these conductive inks is several times lower than the one of metallic films, favouring the use of them in applications where unobtrusiveness of the sensing device is important. However, conductive inks have to be strongly bonded to the underlying substrate, since improper adhesion can result in strain concentrations and ink fracture upon applied deformation on the system. This factor heavily limits also the strategies that can be used to improve the overall stretchability of these systems, since they typically involve deformation in a direction normal to the substrate to increase the deformation allowed before rupture.

The simplest stretchable electronic systems devisable with the materials mentioned above feature a highly deformable substrate, a printed conductive ink such as the ones mentioned above, and surface-mounted devices (SMDs), such as rigid passive components, integrated circuits (ICs), batteries, or connectors. The presence of the rigid components on top of a deformable substrate plays a crucial role in the electromechanical behaviour of these systems, and often there is the need to minimize the dimensions of these ‘rigid islands’ in order to reduce strain concentration happening upon deformation of the system^28–33^. Recent application of this type of strategy, for example, feature the combination of multiple phenomena, such as prestretching the substrate to induce microbuckling or serpentine structures, together with the use of microislands with specific shapes to enhance the system electrical behaviour under cyclic loading^34,35^. However, substrate designs that lead to improvement of the electromechanical behaviour of stretchable systems are also dependent on the complexity on the device itself and on the arrangement of its components, which may strongly influence the local stiffness of the device and its behaviour at high levels of deformation. High levels of prestrain, moreover, may have detrimental effects on the device accurate dimensioning, especially when applied in combination with the creation of stiffer regions on the substrate, which makes the prediction of the device final dimensions far more complex than in the case of a homogeneous system. In order to smoothen the strain transitions in these critical regions, different approaches should be utilised. As seen in a recent review from Xue et al.^36^, the current methodologies used to increase reliability often relay on the use of shielding frames or stiffening substrates right at the rigid component location to limit the strain fields in the vicinities of rigid components^37–41^. However, strain concentration due to the presence of stiffeners and their effect on printed conductive tracks are often taken into account only as a secondary effect of the presence of the stiffener. Moreover, only few of these works focus on strategies to reduce this phenomenon, which promote delamination and premature failure of the system. Other shielding strategies that are currently used rely on open cell substrate to increase breathability and allow to increased deformability of the systems^42–44^.

As seen in previous publications regarding the presence of interconnects on stretchable electronic systems, it is possible to influence local strain through encapsulation and by modifying the geometry of the conductive ink interconnect^8,23^. In addition, vulnerable regions in the substrate, such as areas with rigid-to-soft connections can be reinforced by local enhancement of the stiffness^45,46^. All the cited works, however, mainly refer to increase in system stretchability where the conductive tracks are composed by thin, metallic foils with specific two- or three-dimensional shapes to accommodate high levels of deformation. Currently, very few works focus on the use of printable, electrically conductive inks constituted by conductive Ag-flakes and polymeric matrix, whose mechanical response and electricity conduction diverge strongly from the regular behaviour of pure metallic conductors.

Thus, the aim of this work is to provide and compare suitable strategies to improve the ability of such printed stretchable systems to deform with no destructive effects on the electrical conductivity. In turn, this means to reduce the stress concentrations due to the presence of rigid components, where early failure of the device is often originated. This work presents an approach based on the improvement of electromechanical behaviour of stretchable electronic systems comprising zero-ohm resistors as SMDs through stiffness increase in critical regions. Three fundamental approaches are compared, and critical differences are highlighted:

- Addition of stiffeners on top of SMD components and conductive tracks, defined as *local component encapsulation*; this strategy is very close to the previously proposed framing and micro-island strategies mentioned before.
- Addition of stiffeners in regions around the SMDs, defined as *direct component shielding*.
- Reduction of stiffness of different regions of the system in order to virtually increase the stiffness around the SMD, denoted as *strain dispersion*.

All the strategies above have been thoroughly analysed through FE models and then experimentally tested. The results from the simulations and the experiments were later compared in order to show the details of each strategy and study more in detail the effect of each strategy on the overall behaviour of the stretchable electronic system. Within this context, the reference system will be later mentioned as Reference, while Design 1, 2 and 3 respectively stand for the three strategies mentioned above.

## Results and discussion

**Figure 1:**
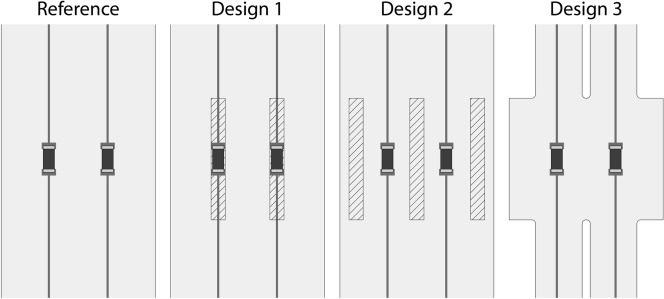
Representation of the different samples tested. The dashed regions represent spots where additional material is placed to increase local stiffness, and the central black components are the SMDs, considered as rigid in the FE models.

The four designs compared for stretchable electronics systems are shown more in detail in Fig. 1, and uniaxial tensile loading was imposed on the different designs up to an overall stretch ratio equal to 1.4. while the imposed boundary conditions and geometrical features are shown in detail in Supplementary Fig. S1. The first sample shown corresponds to a reference sample, used to compare the results coming from the other designs. The second and third samples consist of samples with additional TPU strips that serve to locally increase the stiffness. In particular, Design 1 involves strips placed right under the SMD in order to increase stiffness under the component and wire system in a similar fashion to the well known framing techniques that are extensively employed in literature, in order to enhance the conductive track behaviour right next to the SMD. In Design 2, instead, the additional TPU strips are placed on the sides of the conductive tracks, in order to decrease the stiffener influence on the conductive track behaviour and possible strain concentrations. Design 3 is fabricated by removing materials on other locations of the samples in order to virtually induce a difference in stiffness in the same way of Design 2 and, thus, generate regions with a stiffness lower than the one of the region around the SMD. This process is faster from the fabrication point of view, since it does not involve placement of additional material, but rather the removal of parts of substrate, which can be easily included in normal cutting of the substrate.

**Figure 2:**
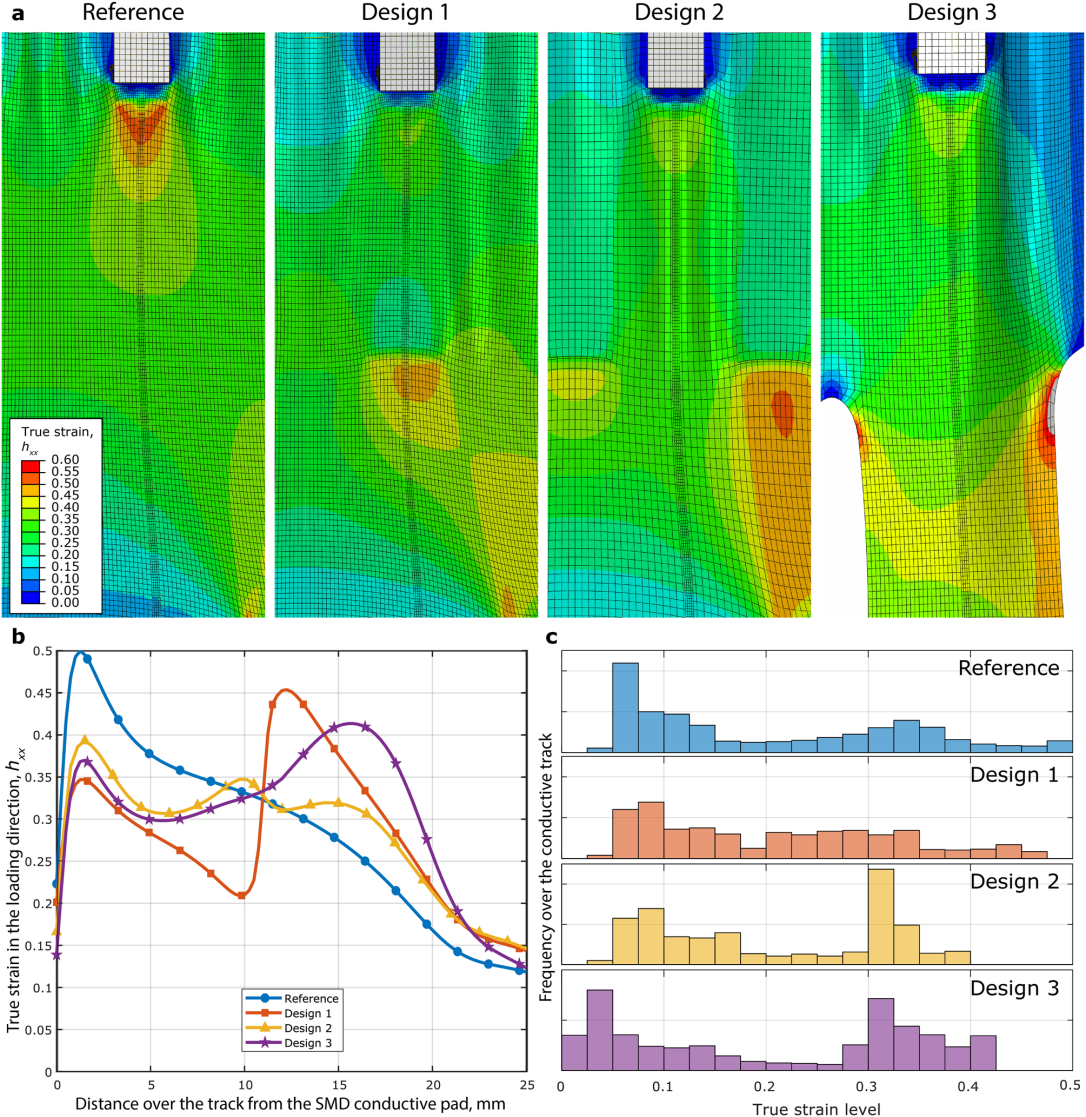
(**a**) Details of the deformed shapes for the different designs around the rigid components; the entity plotted is the true strain in the *x*-direction, which is coaxial with the loading direction. In order to make a clear comparison between the strain states of the different samples at the same overall deformation level, the same colour map was used for the four different cases. (**b**) Plot of the true strain along the *x*-direction along the conductive wire, starting from conductive track beginning near the SMD. It is possible to notice strain concentration in the different designs due to the presence of both the SMDs and the stiffener/holes, depending on the design. (**c**) Histogram plot of the distribution of deformations over the conductive wires.

The mechanical behaviour of the different samples was first analysed using ABAQUS/Implicit; after that, samples were prepared and tested. The results comparison allowed to validate the approach used and helped to maximize the information gathered from a single test. Figure 2a shows some details about the FE results, and, in particular, the effect of the uniaxial deformation imposed on the sample on the deformation fields in the proximity of SMDs. Images in Fig. 2a show the true strain field in the loading direction, ${h}_{xx}$, which is the *x*-direction of Fig. 2. This deformation measure and its relative components are defined according to the relation $\mathbf{h}=ln\mathbf{U}$. These regions are the most critical in terms of the failure of electrical track because of the strain concentration caused by the presence of the rigid component. As can be seen, the tracks reach different levels of maximum deformation depending on the overall geometry and stiffness of the system. In particular, the local true strain on the tracks for the reference sample and Design 1, ${h}_{xx}$, reached local maximum values close to 0.50 due to high concentration of strains due to the rigid component.

Figure 2b shows a comparison of the deformation resulting on the conductive track in the *x*-direction among the different designs. Here it is easier to compare the overall effect of the different shielding methods on the deformation of the conductive track, which results not only in high strain levels in the vicinity of the SMDs, but also strongly depends on the shielding method used. As expected, the highest level of deformation along the track is achieved by the sample without any shielding, while in the region neighbouring the rigid component the other designs show similar levels of deformation for the first 5 mm of the track. However, the deformation levels in Design 1 start to diverge from the other two because of the influence of the stiffener layer, whose edge is located at 11 mm from the beginning of the track. Here it is possible to notice a peak in deformation for Design 1. Deformation levels in Design 2 and 3, instead, increase with a smaller slope in that region. Design 3, in particular, reaches the highest level of deformation along the conductive track direction at roughly 16 mm from the SMD pad, while Design 2 shows a sudden decrease in deformation slightly earlier due to the stiffener on the two sides of the conductive track.

This difference in deformation is a direct result of the different shielding systems. While on one hand all three of these designs have the objective of increasing the system stiffness in the vicinity of the SMDs, the final outcome depends strongly on the strategy applied. As already mentioned, both Design 1 and 2 have similar results in the first part of the track because of the similar method of shielding. In fact the addition of the stiffening TPU strips has a similar effect for the first few millimetres of track in both cases. At a distance of about 5 mm the effect of the position of the additional film starts to increase until, at about 10 mm from the beginning of the track, the presence of its edge right under the track and the sudden decrease of stiffness in that region, results in a local strain amplification in the conductive track direction, as can be seen in Fig. 2b. The presence of the edge of the TPU stiffener results in a sudden increase of the crack density on the conductive track at the boundary of the component and in the creation of possible electrical failure points for the track itself, leading to a scenario similar to the one of the reference design. Design 2, instead, shows none of these sudden peaks in deformation along the track, allowing for a smoother change in deformation along the conductive track. The difference in the maximum levels of true strain reached with the different designs is also shown in Fig. 2c, where histograms of the deformation distributions are shown for the different strategies.

It should be noted that in Designs 1 and 2 very similar procedures are applied in order to improve the electromechanical deformation of the system, and the two samples follow the same fabrication process, that involve the use of additional TPU strips placed in the proximities of SMDs followed by hot press binding of the additional strips. However, the results strongly differ and that is mainly due to the location of the additional stiffener on the substrate. In fact, while in one case its presence interferes and creates additional strain concentration regions on the conductive track, in Design 2 the stiffeners location ensures shielding against excessive deformation levels around the SMD. In this design, however, the stiffener does not directly influence the deformation of the conductive track, and thus does not induce relevant deformation peaks around the SMD. Design 3, instead, follows a different strategy to improve the electrical behaviour under a high deformation of the system: shielding of excessive deformation levels around the SMDs is achieved by decreasing the stiffness in regions that are located away from the SMDs, i.e. by removing material from these locations. The effect of material removal is similar to the final outcome achieved in Design 2, but this strategy offers many advantages under a fabrication point of view, since there is no step added to the production process because cutting is already one of the steps needed. Similar FE analyses were also performed on systems featuring serpentine interconnects and are reported in Supplementary Discussion. These analyses yielded similar results than their counterpart featuring straight interconnects, but also highlighted the need to tailor specific strain limiting strategies to the stretchable system’s geometry and mechanical behaviour.

**Figure 3:**
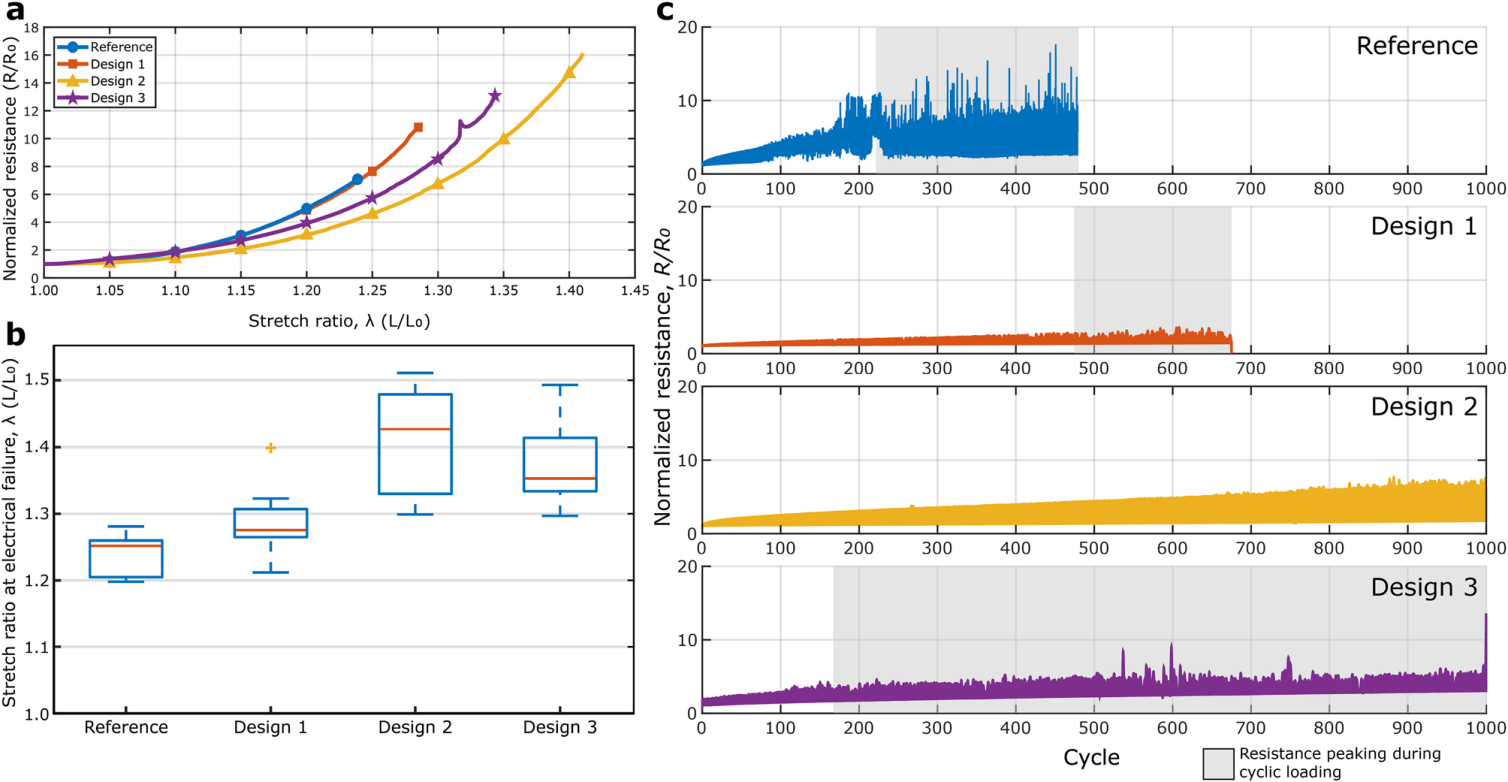
(**a**) Normalized resistance, intended as R/R_0_, of representative samples from each design versus stretch ratio applied. The different designs are shown with different colours, as shown in the legend. (**b**) Box and whiskers plot for the deformation at electrical failure for the different tensile tests performed on the multiple designs. The horizontal orange lines show the average levels of deformation at failure for the different designs, while the boxes and the whiskers represent their quartiles and distribution edges, respectively. The yellow point on design 2 is considered as an outlier for the results. (**c**) Normalized resistance increase upon cyclic loading for the different strategies proposed. The results show how does the resistance increase under a cyclic loading up to λ = 1.1. In particular, the grey areas in the graphs highlight the cycles affected by random resistance peaking upon cyclic loading.

The experimental results well matched with the results obtained from the FE modelling. In fact, the lower strain concentration levels of Designs 2 and 3 also result in a lower overall resistance of the systems under deformation, as shown in Fig. 3a. Here, the evolution of the normalized electrical resistance, calculated as resistance of the deformed sample over the resistance of the undeformed specimen for the most representative samples for each design, $R/{R}_{0}$, is shown with respect to the increase in global strain imposed on the sample. The average initial resistances for the Reference and the three proposed designs were, respectively, equal to 35.4 Ω, 29.3 Ω, 25.5 Ω and 29.3 Ω. It is possible to notice that, while at low deformation levels the resistance build-up develops similarly, starting from a stretch ratio of 1.15 the samples coming from the Design 2 and 3 show a different, and lower, increase in resistance due to the deformation applied. For example, while at a stretch ratio of about 1.2 the samples from Reference and Design 1 show a resistance that is five times the initial one, the specimens coming from Design 2 and 3 show a resistance equal to three times the initial resistance. This difference means that Designs 2 and 3 show a considerably lower change in resistance upon applied deformation, which is equal to 60% of the change in resistance in the reference sample. The same trend continues to higher levels of deformation until failure of the different specimens. The final point of the curves in Fig. 3a is not relative to the electrical failure of the specific samples, but was taken as the average of deformations at the electrical failure point between each set of samples for the different designs, in order to better show the overall behaviour for each of the designs. The results shown follow the trends given by the FE analyses, which is that the samples relative to Reference and Design 1, with similar high peaks and gradients in longitudinal strain along the track for the same level of deformation applied, have a similar resistance evolution with the deformation, and that the same happens for Designs 2 and 3, which also have similar peak levels of deformation.

Electromechanical failure results for the different Designs are shown more in detail in Fig. 3b, where the average deformations at failure for the different designs are collected and shown through a box and whiskers graph. In this case, electrical failure here is defined as the complete loss of conductivity from the sample upon applied stretch ratio. For the sake of clarity, the results graphically represented here are also separately shown as Supplementary Table S1. As expected from the FE analyses, the deformation at failure for the reference sample and Design 1 are similar, even though there is a small increment in electromechanical performance for Design 1. This increment is justified by the lower deformation levels around the SMD, which avoids that failure occurs for delamination of the rigid component, lowering the possibilities of failure. Designs 2 and 3 behave significantly better under deformation, having a deformation at electrical failure at a stretch ratio equal to roughly 1.4. Looking at the deformation results from the FE analyses, this was also expected, noting that the deformation levels for the two specimens are close to each other. The results obtained through cyclic uniaxial loading also follow a similar trend: in fact, for a maximum imposed stretch of 1.1, the reference sample sustained 480 cycles while Design 1 showed complete loss of conductivity at 675 cycles. Designs 2 and 3, instead, both withstood 1,000 cycles without total disconnection. However, as highlighted in Fig. 3c, all the samples except Design 3 showed resistance peaking at high levels of strain starting from very low amounts of cycles. The same samples, however, showed regular levels of electrical resistance right after these peaks and in the adjacent cycles. This peaking can be due to partial delamination of the specimen due to formation of wrinkles on the specimens upon loading. Formation of the wrinkles, moreover, is further emphasized in Design 3, where the cruciform shape allows for complete bending of the sample in its transverse direction during loading, and is believed to cause connection issues at the SMD-conductive track level. Moreover, all designs showed permanent deformation and degradation of the electrical properties to some degree in terms of an increase in baseline resistance. Supplementary Table S2 shows more in detail the increase in base resistance for the different designs.

**Figure 4:**
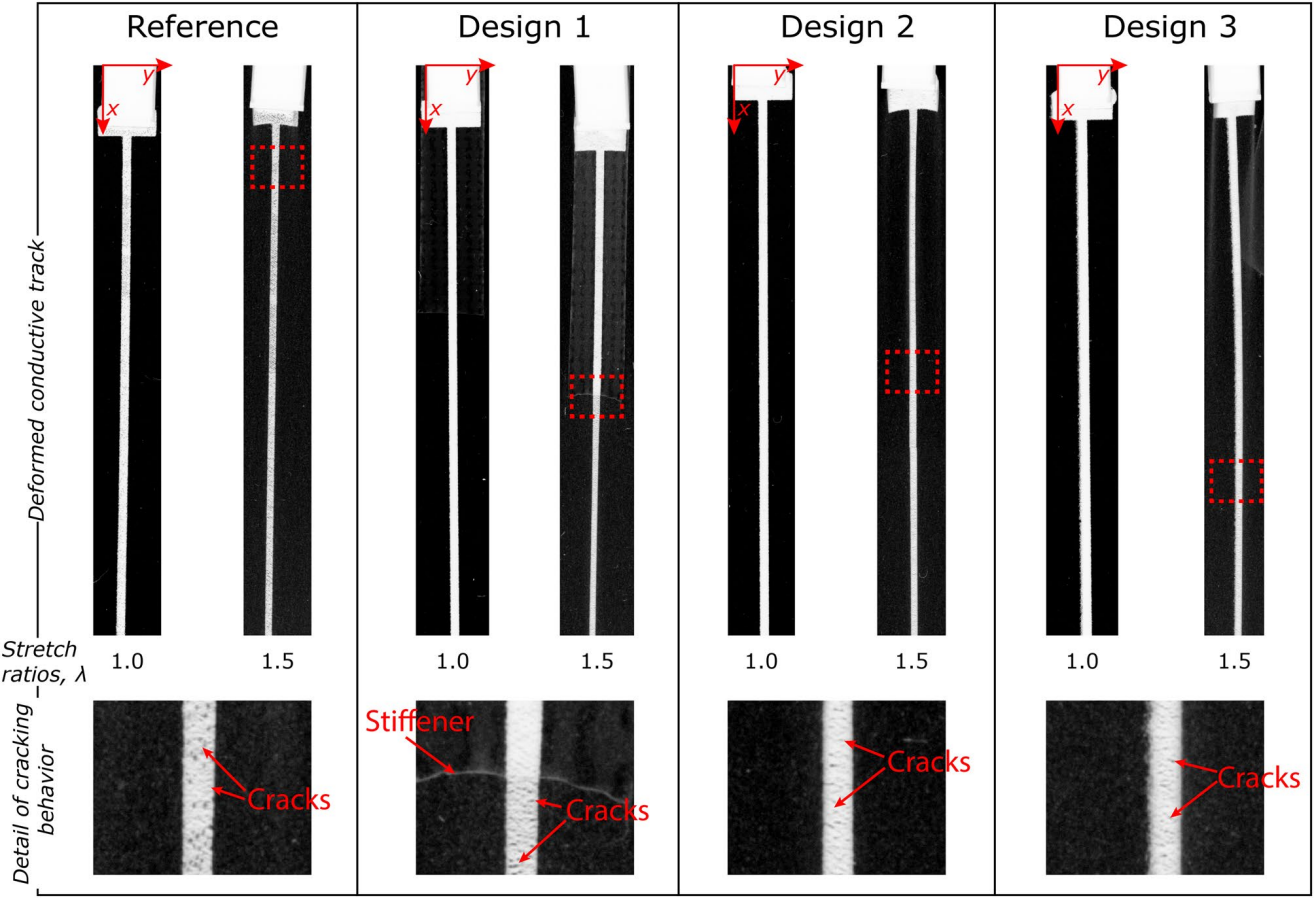
Crack development and deformation of the different samples under tensile deformation in the tracks direction. The images shown are negatives of the original images taken to enhance visibility. It is possible to notice how the different conductive lines deform in different ways depending on the strategies follow to shield regions close to SMDs from excessive deformation. The images were taken at stretch ratios of 1.0 and 1.5, in order to show the cracking evolution on the different designs and to ensure the absence of surface cracks in the undeformed samples. In the figures below there is a detail of the cracking behaviour of each of the four different designs in different locations of the conductive track in order to highlight the differences in mechanical behaviour due to the different geometries.

Stretched samples were also analysed on a system constituted by movable clamps on a backlit table, in order to better analyse the local evolution of crack density on the conductive tracks under tensile loading. Upon the application of uniaxial tension, the samples and the conductive tracks on them deformed similarly as in the FE analysis, with the main difference coming from the ink behaviour. Because of its highly heterogeneous composition, in fact, cracks start to develop in the conductive track as soon as the material is being deformed. This is a typical behaviour for this kind of conductive ink, and occurs because of cracking in the binder matrix, which is the element that actually carries most of the deformation, since the binder has a stiffness that is several times lower the one of the Ag flakes. The development of the cracks has been analysed at different levels of overall deformation of the four designs through photographs of the track with high contrast coming from the presence of the conductive track and of the backlight. Figure 4 shows the behaviour of the different tracks in the undeformed configuration and at a stretch ratio of 1.50. Here it is possible to notice that, as expected from the FE analyses, the local deformation evolves in separate ways for the different samples. In particular, while the reference sample shows a uniform level of deformation, in Design 1 there is a clear separation between the local cracking density on the part of conductive track stiffened by the extra TPU layer and the region without the additional stiffener.

The deformation difference between the two regions generates additional cracking in the conductive track right after the additional layer of stiffener, thus generating a high-resistance region in this spot. The cracking density of Design 2, instead, remains constant with a final crack density that is lower than the one seen in the reference sample. Due to the radical differences in design of Design 3, instead, samples from this design tend to wrinkle more under a tensile deformation. This happens because there are no boundary conditions on the side edges of the sample because of the need to load all designs in similar way. However, these wrinkles occur in regions without conductive tracks and do not affect its cracking behaviour.

**Figure 5:**
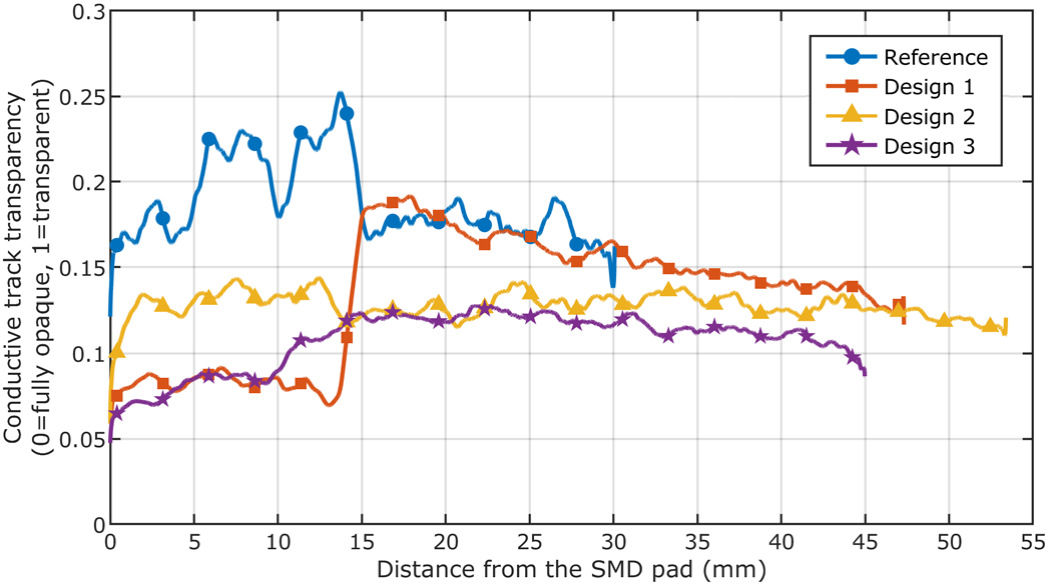
Crack density data obtained from the stretched samples on a backlit table in Fig. 4. Each of the four curves represents the local crack density of the conductive tracks with respect to the distance from the SMD interconnect pad at a total stretch ratio equal to 1.5. For the curve of design 2 it is possible to appreciate the sudden increase in cracking density due to the presence of the edge of the stiffener under the conductive track, which shielded one region from excessive deformation and caused an increase in deformation levels at the end of it.

In order to quantitatively compare this behaviour between the different designs, the registered grayscale images of the tracks at a stretch ratio of 1.5 were extracted and processed by averaging the obtained grayscale values over the y-axes shown in Fig. 4. After that, the obtained curves were smoothed using the MATLAB command ‘smooth’ with the moving average method and a span range of 100 pixels (equal to 1.37 mm of track length) in order to remove excessive noise due to the presence of local cracks and ensure better readability of the results. The obtained values, normalized by dividing the obtained values by 255 because of the 8-bit grayscale original images, are shown in Fig. 5. The normalized values of track opacity, which goes from 0 (completely opaque track) to 1 (fully transparent) can be found to have a correlation with the track axial deformation, as calculated from the FE analyses (as shown in Fig. 3b). This suggests that there is a correlation between crack density in conductive inks and the reached deformation levels. This correlation that is mainly due to the highly heterogeneous composition of the conductive lines, where Ag flakes mechanically behave as rigid objects and the whole deformation has to be sustained by the polymeric binder that keeps the flakes in place. The binder is thus subjected to loads that are several times higher than the overall applied loads and, subsequently, cracks start to develop in the material. However, the different designs develop different cracking densities upon tensile deformation, as the local crack deformation is different for each design. The crack density in the reference sample tends to be higher than in any other design, with some increase in cracking densities for the first 15 mm from the SMD. It is also possible to notice that the behaviour is different for Design 1: in fact, until roughly 15 mm from the start of the track, the additional stiffness provided by the TPU strip ensures a very low cracking density, while after that distance there is a sudden increase in crack density. This behaviour is easily comparable to the local levels of deformation in the deformed track, as shown by the curves in Fig. 3a. The crack density in Design 2, instead, tends to be constant for the whole track length, and it is possible to spot a slow increase in cracking density in Design 3 as the distance from the SMD (and from the stiffer region) increases. Starting at 20 mm from the SMD, the crack density becomes constant also for this design.

This behaviour suggests that, for this class of heterogeneous materials, the relationship between resistance and deformation cannot be written in the form that is widely used for thin metal layers, present in the work from Lu et al.^47^. Moreover, the present work also highlights that the mechanical and conductive behaviour of this class of materials should be considered as strongly different from the one of thin metallic foils, because of the several different features seen in terms of cracking behaviour under deformation and general track behaviour in presence of an SMD. Moreover, the different strategies employed in the three designs analysed also show how several aspects related to mechanical behaviour of the components involved and their geometrical features should be considered when choosing a strategy to improve the overall electromechanical behaviour of stretchable electronic devices. In particular, when upscaling the issue taken into account to more complex electrical devices, featuring several components and with a complex general layout, integration of these strategies gets considerably more complex and a coupled mechanical and electrical design is advised to maximize the efficiency and the device electromechanical properties.

## Conclusions

The need to limit excessive deformation in stretchable electronic systems arises from the need to overcome the current limitations of electronic devices, and many approaches have been adopted in the past in order to improve their electromechanical performance. This work represents an attempt of improving the behaviour of systems that employ printed Ag flake-based conductive inks under tensile loading through three different strategies, each of which is focused on a separate reasoning or fabrication procedure. This was done by first analysing the mechanical behaviour of a stretchable electronic system including, rigid components, a stretchable substrate, and a conductive paste.

Different strategies to improve the overall behaviour of these systems under deformation were then analysed, especially highlighting the behaviour arising from the use of each method. The analysed systems feature printed conductive tracks based on Ag flakes, where stress relieving strategies based on out of plane deformations are not applicable. The approaches represented here show that, even for this kind of stretchable electronic structures it is possible to reduce the resistance change due to the imposed mechanical deformation by stiffening specific regions on the device, thus homogenizing the in-plane strain levels.

In particular, the authors showed that stiffening the region right next to rigid electric components tends to create new sudden gradients in material stiffness, thus concluding that it is generally advisable to employ a different strategy to improve the behaviour under deformations of these systems, since this ‘local stiffening’ may lead to formation of strain concentration regions in other, equally critical parts of the sample. Moreover, different outcomes were achieved using different shielding strategies: in particular, stiffening of regions far enough from the SMD to minimize the influence of stiffness gradients proved to be a useful way of improving electromechanical performance of these systems. In Design 2, this was achieved by equipping the samples with additional strips of TPU at a distance of 15 mm from the SMD in order to avoid influences of stiff regions on the conductive track. In Design 3, instead, material was removed to create regions that were more compliant than the region surrounding the SMD in order to disperse the deformation on the whole sample, and thus away from the rigid component. These two strategies yielded similar results in terms of deformation at electrical failure under tensile loading, while fatigue testing revealed important aspects related to delamination under cyclic loading for these samples. The latter strategy revealed to be more efficient in terms of fabrication process, since cutting is already one of the steps included in the fabrication of stretchable electronics systems. Also, more complex systems, e.g. systems featuring smaller SMDs or a higher amount of them, or even different loading conditions, could benefit from similar procedures featuring solutions that arise from a common ground between solid mechanics and electronics, which stands as a fundamental collaboration for novel development in the field of soft and deformable electronics.

## Materials and methods

### Finite Element analyses

Finite Element (FE) analyses were used to determine the overall shapes for the samples in Fig. 1 using the commercial FE software ABAQUS. In order to properly describe the mechanical interaction between the different materials and their overall behaviour when stretched, each material is modelled as a single material with its own mechanical behaviour. The substrate is defined as an incompressible hyperelastic material following Ogden’s model^48^ with $N=3$, for which the strain energy potential, $\Psi$, is defined as

$$\Psi = \sum_{i=1}^{N}\frac{{\mu }_{i}}{{\alpha }_{i}}\left({\lambda }_{1}^{{\alpha }_{i}}+{\lambda }_{2}^{{\alpha }_{i}}+{\lambda }_{3}^{{\alpha }_{i}}-3\right)$$

The parameters used to describe the substrate behaviour were set equal to the values in Supplementary Table S3, while the terms ${\lambda }_{1}$, ${\lambda }_{2}$ and ${\lambda }_{3}$ are defined as principal stretches, defined as the eigenvalues of the right stretch tensor, $\mathbf{U}$^49^. The same kind of potential, with $N=2$, was used in a previous publication to successfully describe the behaviour of this TPU material^23^. However, Ogden’s model with $N=3$ is generally acknowledged as one of the most widely used for characterization of material behaviour under large deformations^50^.

Due to the arrangement of its constituents and the type of constituents themselves, the conductive ink is described using a linear elastic to bi-linear perfectly plastic behaviour. The employed zero-ohm resistors are instead handled as rigid components in the model. Since the focus of this work is to find ways to optimize the overall geometrical shape of the system to improve its overall electromechanical performance, perfect bonding is considered between the components. The typical mesh size of the substrate part is 0.3 mm and 0.125 mm for the conductive ink, and the element types used for the two components are C3D8H and C3D8, respectively. The difference in mesh sizes between the two parts is needed to analyse in detail the deformation of the conductive ink, and solution convergence was analysed for similarly sized problems in a separate work^51^.

The geometries for the different parts of the models are shown and described more in detail in the supporting information, together with the boundary conditions applied (shown in Supplementary Fig. S1). Since the target is to highlight which strategy is the most suitable to improve the overall behaviour of the system, the initial shape of the sample and the boundary conditions are kept as uniform as possible among the different designs.

### Fabrication of test samples

The authors used a 50 μm thick thermoplastic polyurethane (TPU), U4201 provided by Epurex Platilon as the stretchable substrate, which is provided in rolls and shows a high level of deformation at failure and high dielectric properties. The choice of this substrate ensured that, during tensile testing, there would be no failure occurring because of substrate breakage. A slight (3–5%) prestrain was applied to TPU sheets before attaching them to aluminium plate carrier for the printing process. A highly conductive silver flake paste (CI-1036) from Engineered Conductive Materials (ECM) was employed to fabricate the conductive tracks. The curing temperature of the silver paste (125 °C) is lower than the softening temperature of TPU (155–185 °C); this allows to use this paste on the substrate. Deposition of the silver paste on the substrate was done using a semi-automatic screen printer from TIC (SCF-300). Two consecutive printing cycles were used for fabrication of the conductive tracks. After the printing process, samples were annealed in an oven for 30 min at 125 °C. Samples were then removed from the aluminium plate and some of them (Designs 1 and 2) were partially encapsulated using a heat-press machine (Combo heat press) using the same material as the substrate. In order to evenly compare the results and avoid mismatches in properties due to the application of different fabrication processes, however, the other samples were also subjected to the same process without the addition of an additional layer in specific locations. The heat-press process lasted for 1 min at 150 °C. TopLine Corporation supplied the zero ohm resistors (SR2512), whose physical dimensions are 6.3 mm × 3.2 mm. The recommended pad size for this SMD is 1.6 mm × 3.5 mm. Furthermore, a highly flexible isotropic conductive adhesive (ICA) was used to attach the passive components on the top of the interconnects pads. The ICA, supplied by Namics, is the XE184, a thermoset ICA with silver filler that is typically used for bonding of chip components and connectors.

Samples were annealed in 100 °C for 30 min for the ICA curing. Before the characterization steps, samples were singulated by a laser cutter (Trotec Speedy 100) into the desired template (140 mm × 38 mm). This ensured high dimensional accuracy and good reproducibility of results.

### Samples characterization

An optical microscope was used to ensure adequate resolution of the conductive tracks and to check for any visible defects. Then, initial resistances of samples were measured with a multimeter. The stretchability of interconnects was evaluated through uniaxial quasi-static tensile loading up to total electrical disconnection with an Instron 4411 tensile tester. The uniaxial deformation level imposed on the samples was monitored as stretch ratio, which is defined as $\lambda =L/{L}_{0}$, with $L$ and ${L}_{0}$ respectively equal to the current and reference sample length, in order to keep consistency with the hyperelastic model previously described. Conductivity of the printed tracks can be hindered by applying pressure on them. In order to avoid any damage to the conductive tracks happening for this reason or for the edge effect of the clamp, the clamping system features additional rubber pads that limit the contact between the specimen and the clamping system during gripping. A depiction of the clamping system can be found in Supplementary Fig. S2.

A load sensor of 500 N, pulling speed of 6 mm/min were used for the test. The linking with a Keithley 2425 sourcemeter via a customized software written in LabVIEW for a real-time measurement (sampling frequency of 5 Hz) of the resistance ensured the possibility to monitor the changes in resistance at any moment of the sample stretching under the tensile tester. The interconnects were connected to the Keithley measurement cables using pads of 3M 9703, a pressure-sensitive conductive tape. The schematic of the test setup can be seen from previous works^8^. Sets of 10 samples from each of the four groups were analysed. Moreover, the same setup was also used for cyclic loading of the samples under displacement control. During this kind of tests, the samples were cyclically loaded up to a stretch ratio of 1.1 for 1,000 cycles at a 1 Hz rate, while the resistance increase was measured by using the sourcemeter mentioned above at a sampling frequency of 5 Hz.

Samples’ behaviour under deformation was also separately analysed by uniaxially deforming the sample up to a desired deformation level on a backlit table, in order to get information about the cracking behaviour of the conductive tracks upon loading. This behaviour, expected due to the high heterogeneity of the constituents of the conductive ink employed, was captured using a 5 MP industrial camera together with a Schneider Kreuznach Xenoplan 1:4, a telecentric lens. The use of this lens ensured the exclusion of undesired image aberrations due to perspective distortion and ensures a pixel-to-millimetre ratio equal to 0.0137 for any image taken.

## Supplementary information

Supplementary Information

## Data Availability

The datasets generated during the current study are available from the corresponding author on reasonable request.
