# Supplementary material for: Mechanically driven strategies to improve electromechanical behaviour of printed stretchable electronic systems
Source: Sci Rep. 2020 Jul 21;10:12037. doi: 10.1038/s41598-020-68871-w (PMC7374727; doi:10.1038/s41598-020-68871-w)
Supplement: Supplementary file 1 — Supplementary Information [file 41598_2020_68871_MOESM1_ESM.docx]

Mechanically driven strategies to improve electromechanical behaviour of printed stretchable electronic systems

Donato Di Vito,*^a,b^ Milad Mosallaei, ^b^ Behnam Khorramdel,^b^ Mikko Kanerva^a^ and Matti Mäntysalo ^b^

a. Faculty of Engineering and Natural Sciences, Tampere University, Tampere, Finland.

b. Faculty of Information Technology and Communication Sciences, Tampere University, Tampere, Finland.

*. Corresponding author. E-mail: [donato.divito@tuni.fi](mailto:donato.divito@tuni.fi)

Supplementary information for the article

# Finite Element models employed for the different strategies

The geometries adopted for the different strategies are described here. All the models are constituted by either 3 parts (Reference and Design 3) or 4 parts (Design 1, Design 2). All the parts used are tied together assuming perfect bonding between them, thus excluding any possibility of delamination.

The geometries employed to model the behaviour of the different layouts are shown in Supplementary Figure S1, where also the applied boundary conditions (BCs) are included. In order to reduce the computational cost of each simulation and because of the sample and loading condition symmetry, the models are a quarter of the real samples, and consequently symmetry constraints are applied on two of the sample sides. Moreover, a total displacement of 10mm was applied on each of the designs modelled, similarly to a nominal strain of 0.4 imposed on the specimens. As described also in the main text, the substrate and the conductive ink were modelled, respectively, as an incompressible hyperelastic material and a linear elastic to perfectly plastic material. More details about the parameters used to describe the material behaviour are given in detail in Supplementary Table S2. The typical mesh size of the substrate part is 0.3 mm and 0.125 mm for the conductive ink, and the element types used for the two components are C3D8H and C3D8, respectively. The difference in mesh size arises from the need to investigate in detail the deformation fields of the conductive ink layer, and different mesh sizes were thoroughly investigated to check for convergence of the solutions in similar sized problems in a different work, as described in the Materials and Methods section of the main text.


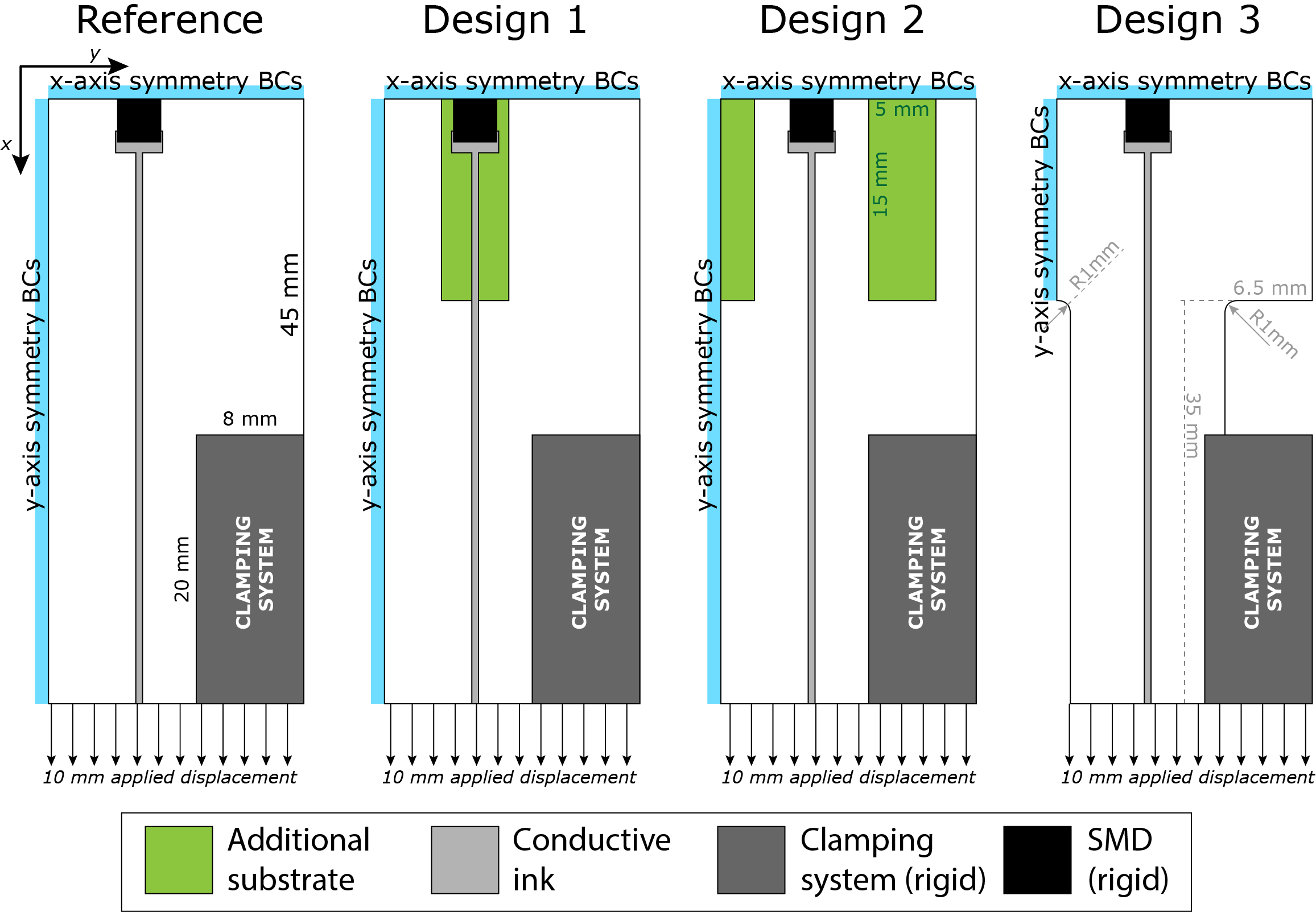
Supplementary Figure S1 - Dimensions and applied BCs for the different designs. The black outlines denote the basic shape of the system, which corresponds to the reference sample, while the other lines correspond to the designs mentioned in the legend. The main boundary conditions of the system are the two symmetry constraints, indicated with cyan colouring, and the arrows pointing downwards indicate the applied displacement on the sample side. As shown in figure 2, a displacement equal to 10 mm was applied on one of the sides in order to mimic uniaxial tension.

Supplementary Table S1 - Deformation at failure for the different designs. The listed values are given for the different designs as stretch ratios applied at the point of electrical failure.

|  | **Stretch ratio at failure for the different designs** | | | | |
| --- | --- | --- | --- | --- | --- |
|  | **Mean** | **Min** | **Max** | **Lower quartile** | **Higher quartile** |
| **Reference** | 1.240 | 1.198 | 1.281 | 1.205 | 1.260 |
| **Design 1** | 1.276 | 1.212 | 1.323 | 1.265 | 1.307 |
| **Design 2** | 1.427 | 1.299 | 1.511 | 1.330 | 1.479 |
| **Design 3** | 1.353 | 1.297 | 1.493 | 1.334 | 1.414 |

Supplementary Table S2 – Resistance baseline increase at different cycles through the cyclic uniaxial tests.

|  | **Resistance at the cycle number** | | | |
| --- | --- | --- | --- | --- |
|  | **1** | **480** | **675** | **1000** |
| **Reference** | 19.74 Ω | 45.79 Ω | **Failed** | **Failed** |
| **Design 1** | 17.14 Ω | 22.31 Ω | 23.67 Ω | **Failed** |
| **Design 2** | 14.20 Ω | 18.67 Ω | 22.08 Ω | 24.56 Ω |
| **Design 3** | 21.87 Ω | 43.23 Ω | 48.66 Ω | 56.67 Ω |

Supplementary Table S3 - Mechanical properties of the different components of the systems modelled in the FE analyses.

| **TPU properties** | | | |
| --- | --- | --- | --- |
| $\boldsymbol{i}$ | $\boldsymbol{\mu}_{\boldsymbol{i}}$ | | $\boldsymbol{\alpha}_{\boldsymbol{i}}$ |
| **1** | -4.725 | | 1.402 |
| **2** | 1.392 | | 3.295 |
| **3** | 9.196 | | -2.075 |
| **Conductive paste properties** | | | |
| $\boldsymbol{E}$ **(GPa)** | | 1.725 | |
| $\boldsymbol{\nu}$ | | 0.3 | |
| **Yield stress 1 (MPa)** | | 19.67 at $\varepsilon_{P}=0$ | |
| **Yield stress 1 (MPa)** | | 24.35 at $\varepsilon_{P}=0.0698$ | |


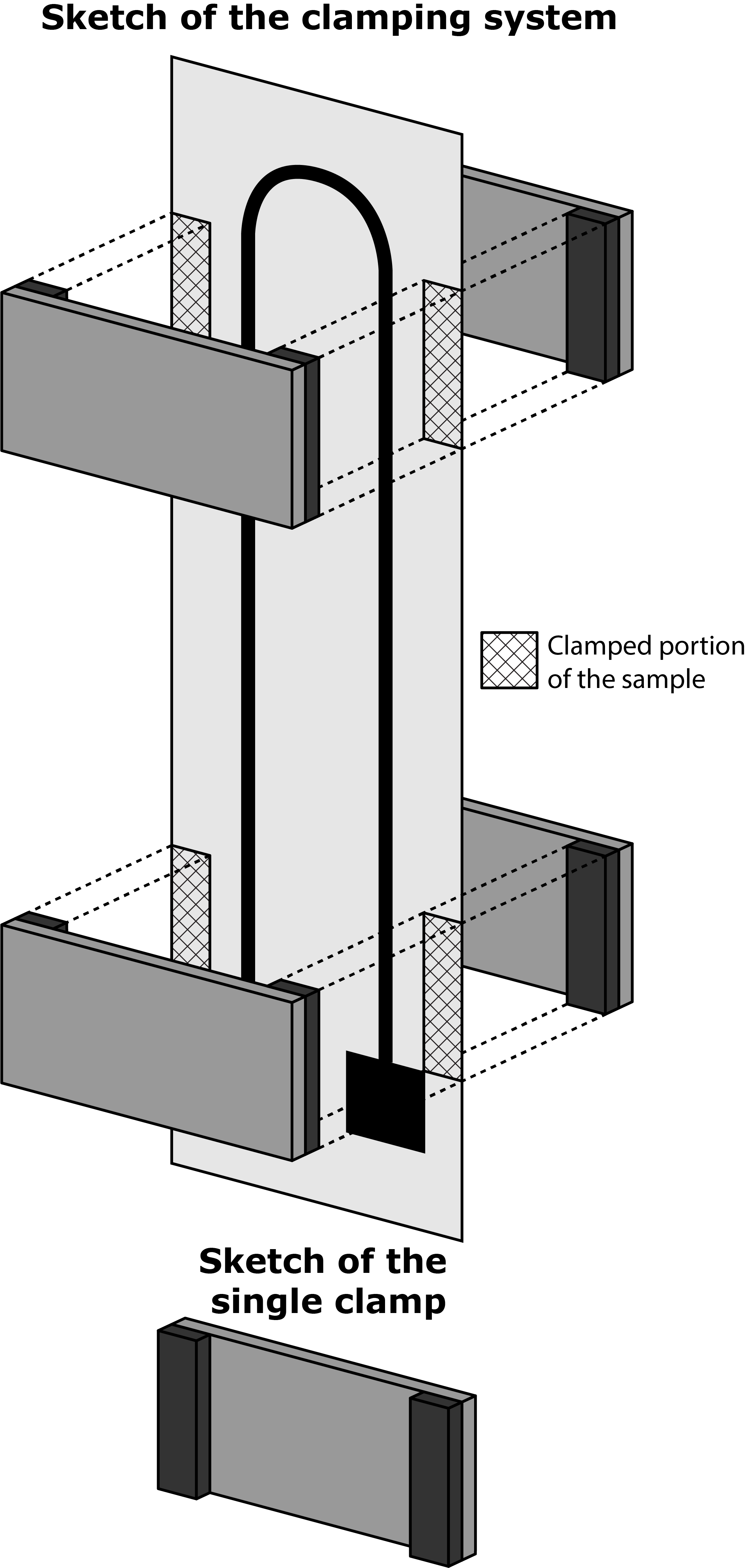
Supplementary Figure S2 – Depiction of the clamping system for tensile testing and of the single clamp, composed of a rigid plate having the same width as the sample and of two rubber pads that allowed to clamp only the edges of the specimen, without interfering with the printed conductive tracks.

# Supplementary Discussion: Application of the same strain limitation strategies on serpentine interconnects

Serpentine interconnects are widely used to reduce strain concentrations on stretchable conductive systems. Several authors have studied the optimal geometry of this kind of interconnects to minimize the deformation occurring because of tensile load on these structures; some examples are the works from Widlund et al. (2014) Zhang et al. (2013) and Gonzalez et al. (2008), already mentioned in the main document. Using the same structures proposed in the main text, the authors analysed the improvements in terms of strain peaks limitation due to their application in systems employing serpentine interconnects.


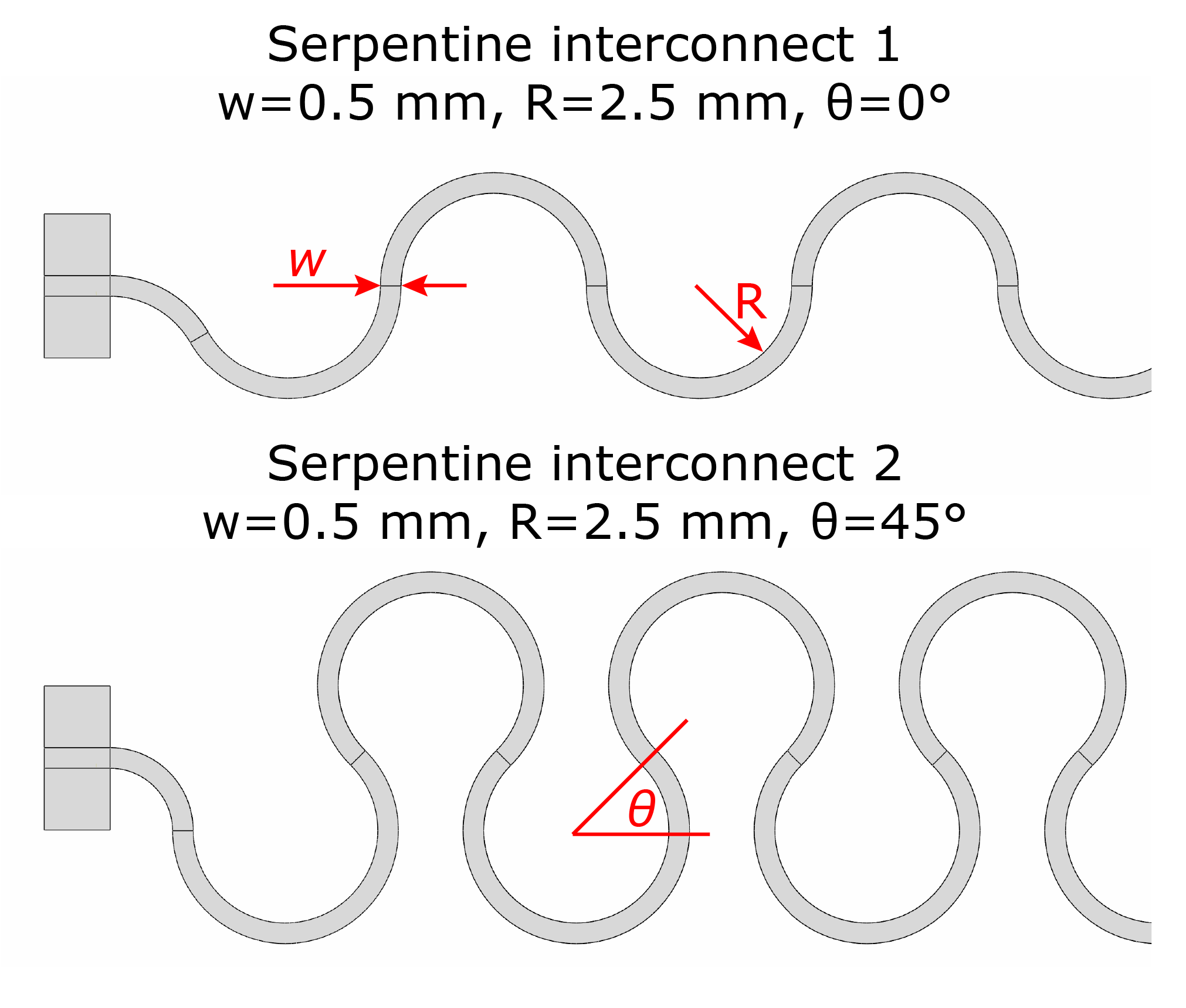
Two different geometries were chosen to compare their FE results. While the track width was kept constant and equal to the one employed with the straight interconnects (equal to 0.5mm), a radius equal to 2.5mm was chosen for the meanders (thus yielding a R/w parameter equal to 5) and two different arc angles θ were chosen, 0° and 45°. The resulting geometries are shown in figure S3.

Figure S3 – Designs used for the serpentine interconnects.

The straight interconnects, then, were substituted with the serpentine interconnects created and the FE results were generated using the same boundary conditions of the systems with straight lines, and the results are shown in Figures S4 and S5, respectively for θ equal to 45° and 0°. For θ=0°, all the designs are shown to contribute to the decrease of strain concentration near the SMDs, although the trend here is different from the one in the straight interconnects geometry. Designs 1 and 2, in fact, show a closer behaviour than before while Design 3 is actually the one showing the highest levels of deformation along the conductive tracks, as can be seen from figure S4b. A similar behaviour is also encountered with the serpentine interconnect having θ=45°, where the strain peaks for the different strategies are even more amplified. This happens because, while in this case the conductive track width is equal to 0.5 mm, the ‘effective’ interconnect width, i.e. the total width occupied by the track geometry, is far wider than in the straight interconnect case; in fact, while the track width and the effective interconnect width are the same, the two geometries proposed have an effective interconnect width equal to 5.5 mm for θ=0° and 9.05 mm for θ=45°. This means that, when using these designs for the interconnects, it is necessary to consider strain concentrations developing over a wider region of space. This reinforces the concept that these three strategies need to be designed ad hoc for the intended system design, and that the variation of one parameter could heavily interfere with the mechanical behaviour of the whole system. As an example, figure S6 shows the difference in


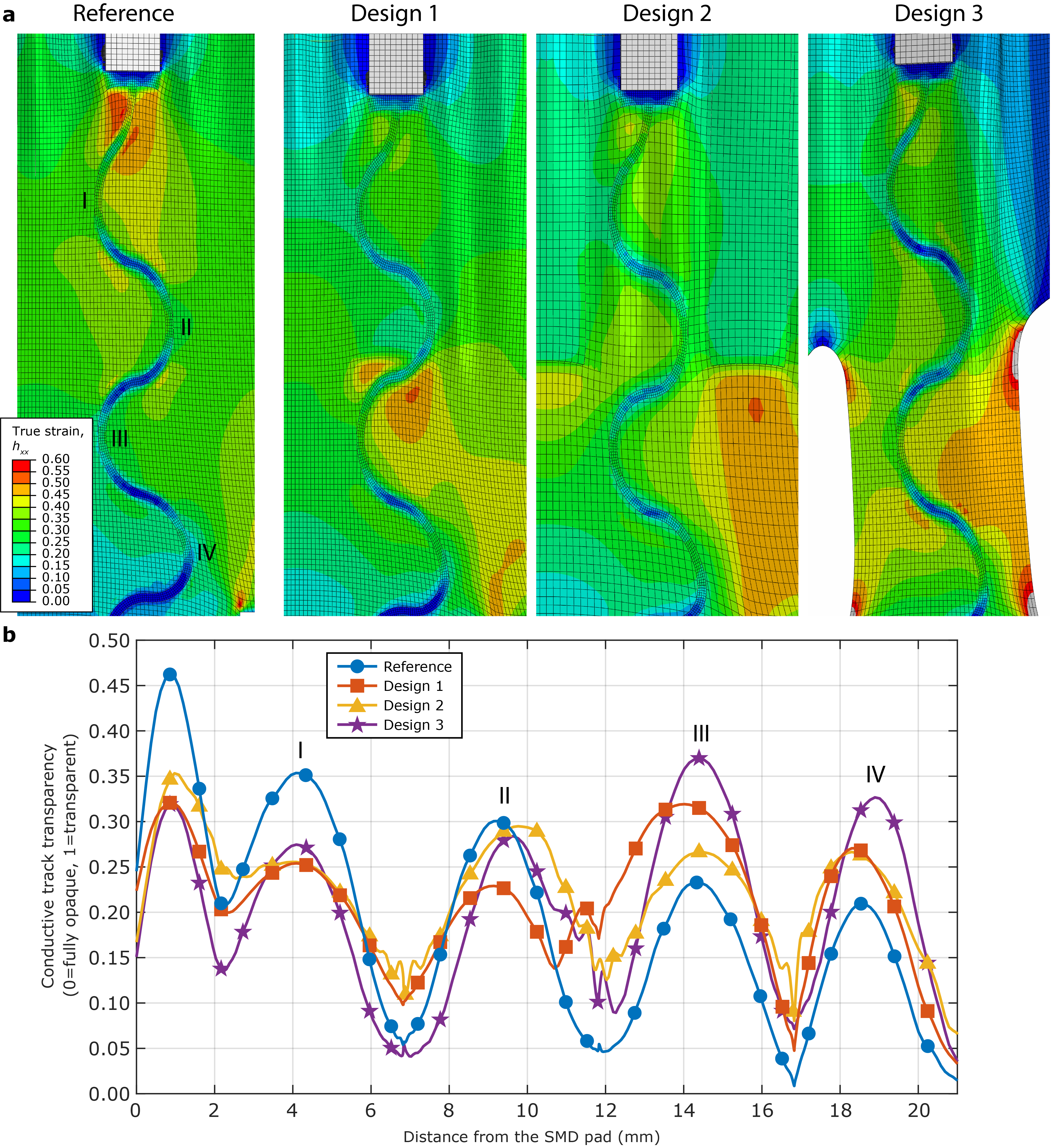
Supplementary Figure S4 – Application of the designs proposed in the work on samples featuring serpentine interconnects with θ=0°. Although the strategies have a different influence than in the straight interconnect geometry, it is still possible to notice their beneficial effect on the strain field of the sample.

results that can be obtained by moving the cutting line in Design 3 0.5 and 1 mm away from the interconnect. It is possible to notice that the strain field on the sample is modified by the geometry of the removed material. This results in a notable change in the strain field on the serpentine interconnect, as shown in the graph of figure S6, and in the maximum strain along the track sensibly decreasing as the sample border gets further from the interconnect, thus reducing the strain concentrations due to the presence of the edge on the interconnect.


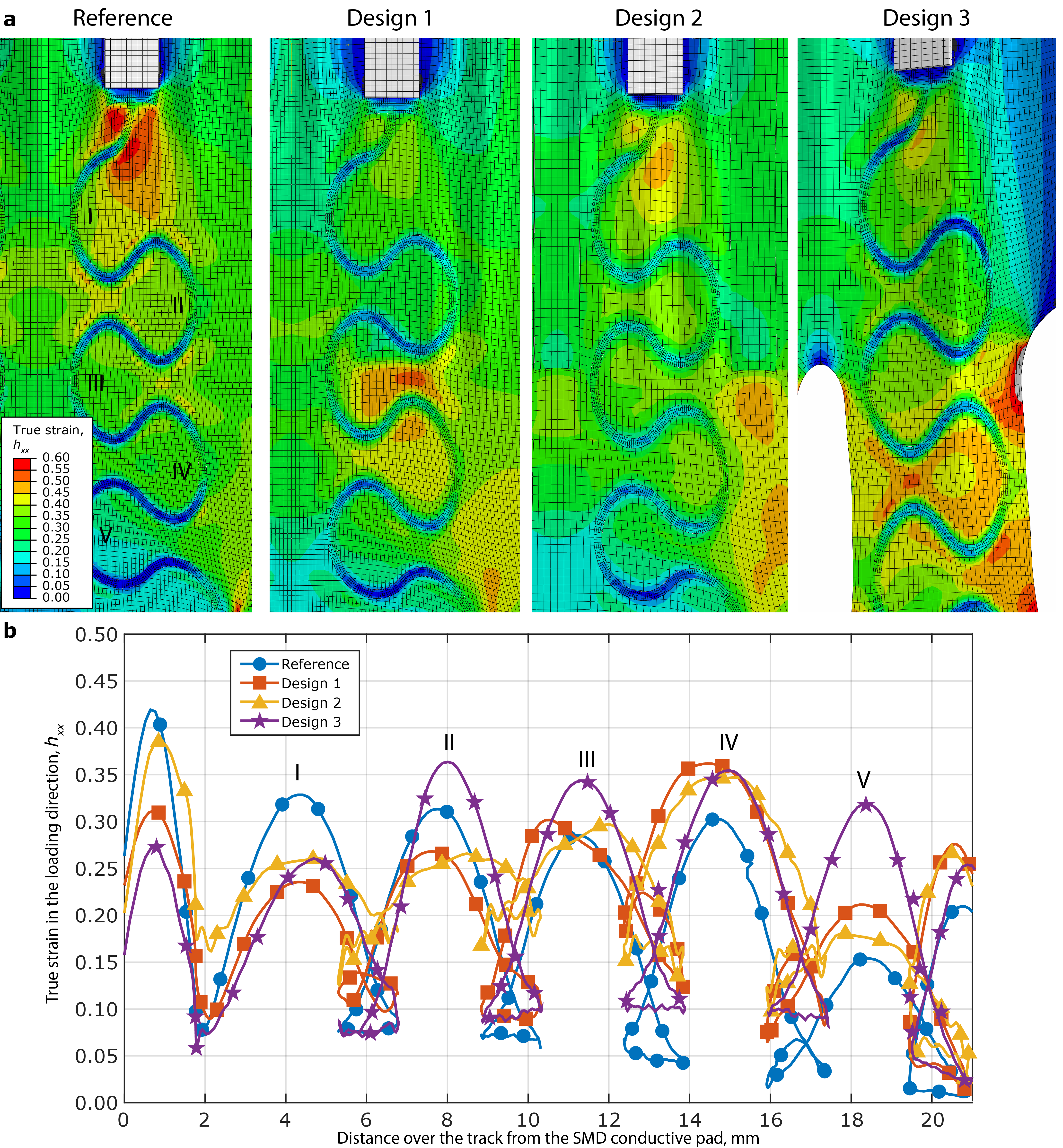
Supplementary Figure S5 – Application of the designs proposed in the work on samples featuring serpentine interconnects with θ=45°. Although the strategies have a different influence than in the straight interconnect geometry, it is still possible to notice their beneficial effect on the strain field of the sample.


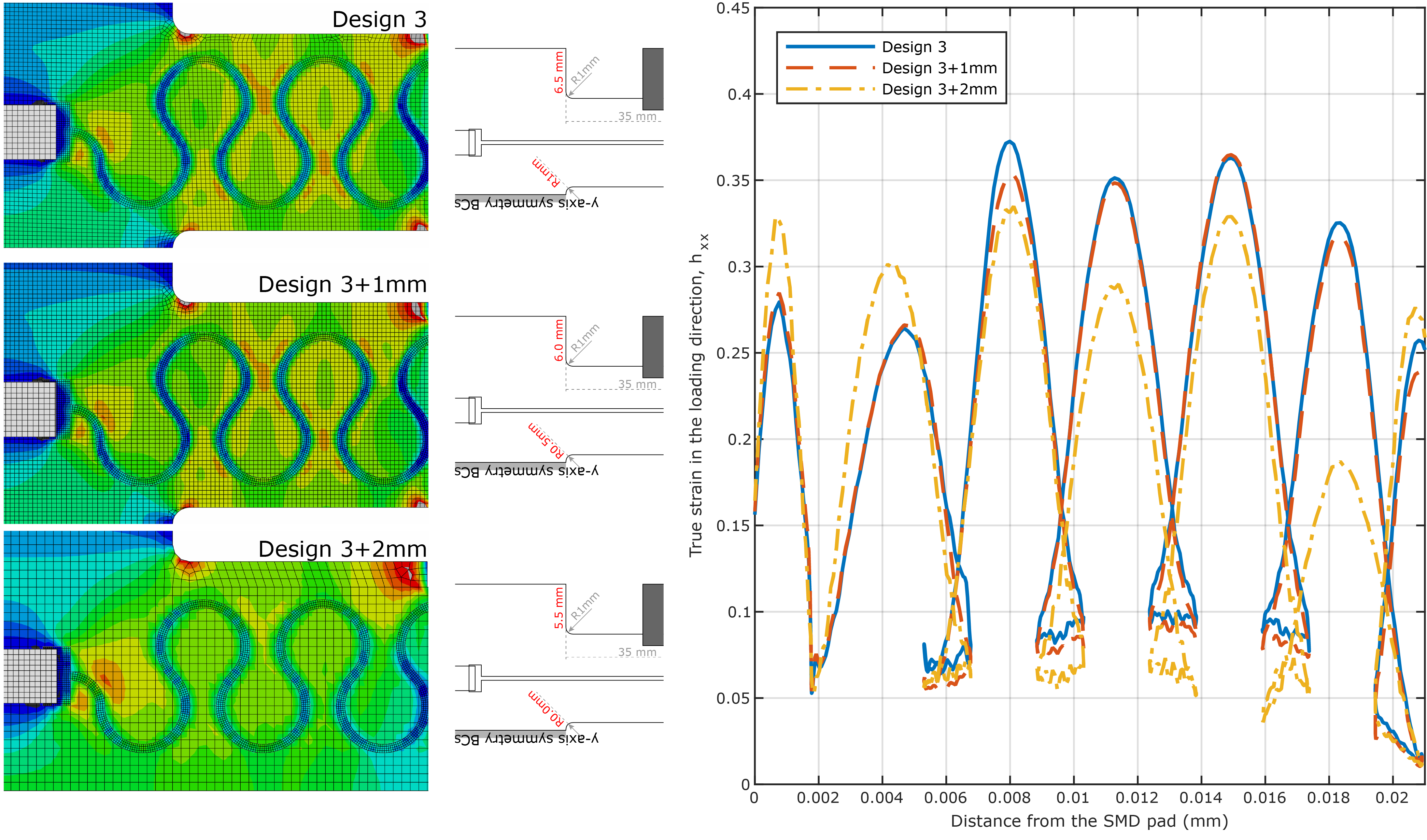
Supplementary Figure S6 – Analyses of the changes in mechanical behaviour of the stretchable electronic sample through modification of the geometry of Design 3. It is possible to notice how the strain field on the sample changes as the two parameters in red in the picture are being changed. The graph on the right shows the strain field on the serpentine conductive track versus the distance from the SMD pad.
